# Caregiver-reported health-related quality of life of New Zealand children born very and extremely preterm

**DOI:** 10.1371/journal.pone.0253026

**Published:** 2021-06-08

**Authors:** Gordon X. H. Liu, Jane E. Harding, for the PIANO Study Team

**Affiliations:** Liggins Institute, University of Auckland, Auckland, New Zealand; University of New South Wales, AUSTRALIA

## Abstract

**Background:**

Children born preterm, particularly at earlier gestations, are at increased risk for mortality and morbidity, but later health-related quality of life (HRQoL) is less well described. Neurodevelopmental impairment and socio-economic status may also influence HRQoL. Our aim was to describe the HRQoL of a cohort of New Zealand children born very and extremely preterm, and how this is related to neurodevelopmental impairment, gestational age, and socio-economic deprivation.

**Methods:**

Children born <30 weeks’ gestation or <1500 g birthweight were assessed at 7 years’ corrected age. Caregivers completed the Child Health Questionnaire Parent Form (CHQ-PF50), and the Health Utilities Index Mark 2 (HUI-2). Neurodevelopmental impairment was defined as Wechsler full scale intelligence quotient below -1 standard deviation (SD), Movement Assessment Battery for Children total score ≤15 percentile, cerebral palsy, deafness, or blindness.

**Results:**

Data were collected for 127 children, of whom 60 (47%) had neurodevelopmental impairment. Overall, HRQoL was good: mean (SD) CHQ-PF50 physical summary score = 50.8 (11.1), psychosocial summary score = 49.3 (9.1) [normative mean 50 (10)]; HUI-2 dead-healthy scale = 0.92 (0.09) [maximum 1.0]. Neurodevelopmental impairment, lower gestational age, and higher socio-economic deprivation were all associated with reduced HRQoL. However, on multivariable analysis, only intelligence quotient and motor function were associated with psychosocial HRQoL, while intelligence quotient was associated with physical HRQoL.

**Conclusions:**

Most seven-year-old children born very and extremely preterm have good HRQoL. Further improvements will require reduced neurodevelopmental impairment.

## Introduction

Health-related quality of life (HRQoL) can be defined as “health state… which reflects the physical, psychological, social, and emotional well beings” [1]. It is now regarded as an important outcome of medical practice and research as it provides a holistic understanding of participant health.

Babies born very and extremely preterm make up 15.6% of total preterm births [2]. Although survival rates have improved steadily, these babies still experience significant morbidity and mortality relative to their full-term peers.

Neurodevelopmental impairment (NDI), such as reduced intelligence and cerebral palsy, is associated with reduced HRQoL in childhood [3, 4]. Earlier gestation at birth and increased socio-economic deprivation are also associated with reduced HRQoL [5, 6]. However, NDI is also associated with earlier gestation at birth and increased socio-economic deprivation [7, 8], and socio-economic deprivation is itself a risk factor for preterm birth [9]. Thus, it remains unclear which of these factors is most important for later HRQoL.

To our knowledge, there are no reports describing HRQoL in New Zealand children born very or extremely preterm. Therefore, we aimed to describe the HRQoL of a cohort of New Zealand children born very and extremely preterm, and determine the relationships between HRQoL and NDI, gestation at birth, and socio-economic deprivation.

## Materials and methods

The Protein, Insulin and Neonatal Outcomes study comprised a subgroup of children born <30 weeks’ gestation or <1500 g birthweight and admitted to the neonatal intensive care unit at National Women’s Health, Auckland from 2005–2008, who had participated in previous neonatal studies (for cohort details see [10]). Participants underwent a multidisciplinary assessment at 7 years’ corrected age, including the Wechsler Intelligence Scale for Children 4^th^ Edition (WISC-IV) and Movement Assessment Battery for Children 2^nd^ Edition (MABC-2). Visual acuity was assessed using a crowded logMAR chart. Cerebral palsy (CP) was diagnosed by neurological examination carried out by a pediatrician using a standard definition [11], and severity graded using the Gross Motor Function Classification Score (GMFCS) [12].

Caregivers completed the Child Health Questionnaire Parent Form 50 Questions (CHQ-PF50) and the modified Health Utilities Index Mark 2 (HUI-2) excluding the “Fertility” attribute [13–15]. Both measures of HRQoL assess physical, mental, and emotional wellbeing in children, with the CHQ-PF50 also assessing the child’s impact on family dynamics, and the HUI-2 also reporting on cognitive outcomes. All domains in the CHQ-PF50 are scored on a 0–100 scale, except for the “Change in health” domain which is scored on a 0–1 scale. To assess overall HRQoL, the CHQ-PF50 derives a physical summary score describing physical HRQoL, such as whether the child can mobilize freely or without pain, and a psychosocial summary score describing psychosocial HRQoL, such as whether the child has good self-esteem or is free from anxiety. In a normative reference population, both the physical and psychosocial summary scores have a mean of 50 and a standard deviation (SD) of 10 [13]. The HUI-2 produces a “Dead-healthy scale”, with all domains in the HUI-2 being scored from 0–1. For both questionnaires, 0 represents the worst possible health state and the upper score represents the best.

We used NZDep2013 as a measure of socio-economic deprivation, which is a 1–10 decile scale, with higher values denoting greater deprivation [16]. Ethnicity was prioritized in the following order: Māori, Pacific, Asian, European or Other [17].

Ethics approval for the Protein, Insulin and Neonatal Outcomes study was obtained from the New Zealand Health and Disability Ethics Committee (NTY/12/05/035) and the Auckland District Health Board Research Review Committee (ADHB 5486). Informed written consent was obtained from the participant’s caregiver(s) before beginning the assessment, and verbal assent was sought from the participant.

### Definitions

NDI was defined as having any of the following: WISC-IV full-scale intelligence quotient (FSIQ) more than 1 SD below the population mean (i.e. <85); MABC-2 total score ≤15 percentile; cerebral palsy; hearing impairment requiring hearing aids; or visual acuity of 6/60 (1.0 logMAR) or worse in the better eye.

Mild NDI was defined as having WISC-IV FSIQ between 1 and 2 SD below the population mean (i.e. 70–84), or MABC-2 total score between the 5^th^ to ≤15^th^ percentile. Severe NDI was defined as having any of the following: WISC-IV FSIQ below -2 SD (i.e. <70); MABC-2 total score ≤5 percentile; cerebral palsy; hearing impairment requiring hearing aids; or visual acuity of 6/60 (1.0 logMAR) or worse in the better eye.

Reported HRQoL measures were compared between children with and without NDI, different gestation at birth (above or below the median of 26 completed weeks), and different socio-economic status (NZDep2013 ≤ or >5).

### Statistical analysis

Data were analyzed using JMP 15.0.0® (SAS Institute Inc., Cary, NC, USA), and are presented as n (%), mean (SD), or median (interquartile range). Continuous data were assessed for normality and compared between groups using two-tailed between-subjects t-tests or one-way ANOVA if normally distributed, with Tukey’s *post hoc* adjustment for multiple comparisons. The outcome “Parental impact–emotional” from the CHQ-PF50 was square-root transformed to a near-normal distribution. The remaining data were analyzed using the Mann-Whitney U test or Kruskal-Wallis ANOVA, with Steel-Dwass *post hoc* analysis.

Regression analyses were used to explore potential independent predictors of overall HRQoL. Univariable analyses were run between WISC-IV FSIQ, MABC-2 total score percentile, gestational age, and NZDep2013 against overall HRQoL (physical summary score, psychosocial summary score, and dead healthy scale score). Variables with p <0.15 were then included in multivariable analysis. Collinearity was defined as variance inflation factor >10 and interaction effects were not assessed.

The level of significance was defined as 0.05.

## Results

**Fig 1:**
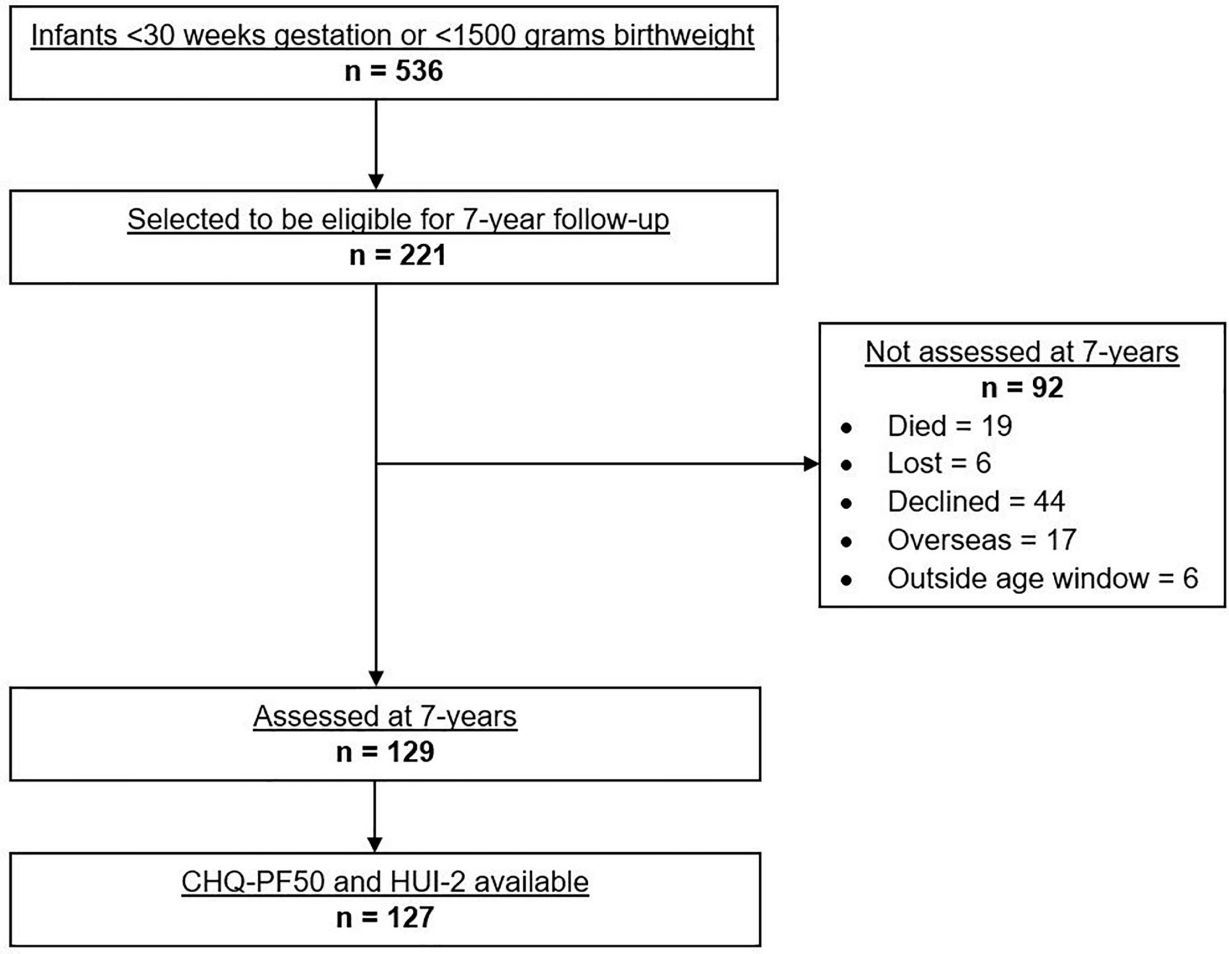
Flow diagram of participants in this follow-up study. CHQ-PF50: Child Health Questionnaire Parent Form 50 questions. HUI-2: Health Utilities Index Mark 2.

Of the 221 children who were selected to be potentially eligible for participation in this follow-up study, 129 (58%) were assessed at 7-years (Fig 1). CHQ-PF50 and HUI-2 data were available for 127 participants, half of whom were boys and 72% were singletons (Table 1). Nearly half (44%) were Māori or Pacific Islander, and a further 14% were Asian. One-quarter lived in highest deprivation areas. Mean gestational age was 26.7 weeks, and 6% (7/124) had a diagnosed neurological insult during their neonatal course (either intraventricular hemorrhage grades 3 or 4, periventricular leukomalacia, or both). At 7 years, 47% were categorized as having NDI, with the majority having an FSIQ <85 (32%), an MABC-2 total score ≤15 percentile (35%), or both (21%).

**Table 1:** Participant demographics.

	N = 127
Boys	67 (52.8)
Singletons	92 (72.4)
Gestational age (weeks)	26.7 (1.9)
Birth weight (g)	920 (230)
Birth weight z-score	0.04 (0.9)
Birth length (cm)	34.6 (2.9)
Birth length z-score	-0.04 (1.1)
Birth head circumference (cm)	24.6 (2.0)
Birth head circumference z-score	0.20 (1.1)
Any antenatal steroids	115 (90.6)
Preterm pre-labour rupture of membranes†	91 (71.7)
Chronic lung disease†	39 (30.7)
Necrotizing enterocolitis†	5 (3.9)
Early infection†	1 (0.8)
Late infection†	23 (18.1)
Intraventricular hemorrhage grades ¾†	7 (5.6)
Periventricular leukomalacia†	4 (3.3)
Retinopathy of prematurity grades ¾†	15 (12.4)
Neurodevelopmental impairment	60 (47.2)
Mild neurodevelopmental impairment	38 (29.9)
Severe neurodevelopmental impairment	22 (17.3)
WISC-IV full scale IQ	90.0 (16.2)
WISC-IV full scale IQ <85	41 (32.3)
WISC-IV full scale IQ <70	10 (7.8)
MABC-2 total scaled score	8.0 (3.7)
MABC-2 total score ≤15^th^ percentile	44 (34.6)
MABC-2 total score ≤5^th^ percentile	34 (26.8)
WISC-IV full scale IQ <85 and MABC-2 total scaled score ≤15^th^ percentile	26 (20.5)
Cerebral palsy	10 (7.9)
Hearing impairment requiring hearing aids	2 (1.6)
Visual impairment	0 (0.0)
New Zealand Deprivation Index 2013 quintiles	
1	23 (18.1)
2	26 (20.5)
3	21 (16.5)
4	23 (18.1)
5	34 (26.8)
Ethnicity	
Māori	37 (29.4)
Pacific	18 (14.3)
Asian	18 (14.3)
European or Other	53 (42.1)

Values are n (%) or mean (SD). Birth length n = 119. Birth length z-score n = 118. Birth head circumference n = 123. Birth head circumference z-score n = 122. Intraventricular hemorrhage grades ¾ n = 124. Periventricular leukomalacia n = 120. Retinopathy of prematurity n = 121. Participant ethnicity n = 126. Visual impairment: best visual acuity <6/60. WISC-IV: Wechsler Intelligence Scale for Children 4^th^ edition. MABC-2: Movement Assessment Battery for Children 2^nd^ edition. IQ: Intelligence quotient.

†Defined according to the Report of the Australian and New Zealand Neonatal Network 2011 [18].

Ten children were diagnosed with CP, four with a GMFCS of 1, three with a GMFCS of 2, one with a GMFCS of 5, and one without any functional impairment. Nine of these ten children had MABC-2 percentile ≤5. When children with CP were excluded, the cohort mean (SD) MABC-2 total scaled score increased slightly to 8.5 (3.1).

When participants were categorised according to NDI, gestational age, and socio-economic deprivation, there were more participants with NDI in the more deprived than the less deprived group, although the difference was not statistically significant (37/68, 54% vs 23/59, 40%, p = 0.08). There were similar numbers of participants born at earlier gestations in the more deprived and less deprived group (36/68, 53% vs 31/59, 53%, p = 0.96), and of participants with NDI in the earlier gestation and later gestation groups (30/67, 45% vs 30/60, 50%, p = 0.56).

### CHQ-PF50

Overall, caregivers reported that participants had good HRQoL, with both physical and psychosocial summary scores averaging 50 (Table 2). Across individual domains, those pertaining to physical health were scored the highest: participants experienced little to no pain, could perform most physical tasks, and could freely engage in social activities. Families generally got along well, with participants rarely interrupting activities, restricting time for caregiver self-care, or behaving disruptively. Caregivers also reported good mental health outcomes for participants, describing them as largely unaffected by self-esteem issues or feelings of anxiety or depression.

**Table 2:** Health-related quality of life scores for children with and without neurodevelopmental impairment, lower gestation, and greater socio-economic deprivation.

Domain	Overall cohort score		Subgroup Scores		
		Risk factor	Yes	No	p
**Child Health Questionnaire Parent Form 50 questions**					
Physical functioning†	100.0 (88.9, 100.0)	Neurodevelopmental impairment	100.0 (79.2, 100.0)	100.0 (94.4, 100.0)	0.007
		≤26 weeks’ gestation	100.0 (77.8, 100.0)	100.0 (94.4, 100.0)	0.035
		More deprived	100.0 (83.3, 100.0)	100.0 (94.4, 100.0)	0.040
Social limitations—emotional†	100.0 (77.8, 100.0)	Neurodevelopmental impairment	88.9 (44.4, 100.0)	100.0 (100.0, 100.0)	<0.001
		≤26 weeks’ gestation	100.0 (55.6, 100.0)	100.0 (88.9, 100.0)	0.029
		More deprived	100.0 (66.7, 100.0)	100.0 (88.9, 100.0)	0.012
Social limitations—physical†	100.0 (100.0, 100.0)	Neurodevelopmental impairment	100.0 (66.7, 100.0)	100.0 (100.0, 100.0)	0.002
		≤26 weeks’ gestation	100.0 (83.3, 100.0)	100.0 (100.0, 100.0)	0.124
		More deprived	100.0 (83.3, 100.0)	100.0 (100.0, 100.0)	0.197
Bodily pain/discomfort†	100.0 (80.0, 100.0)	Neurodevelopmental impairment	100.0 (72.5, 100.0)	100.0 (80.0, 100.0)	0.602
		≤26 weeks’ gestation	100.0 (80.0, 100.0)	100.0 (70.0, 100.0)	0.315
		More deprived	100.0 (80.0, 100.0)	100.0 (80.0, 100.0)	0.376
Behavior	70.7 (18.1)	Neurodevelopmental impairment	65.3 (19.1)	75.5 (15.7)	0.001
		≤26 weeks’ gestation	70.3 (19.2)	71.0 (17.0)	0.830
		More deprived	69.0 (19.4)	72.6 (16.4)	0.260
Mental health	77.8 (12.6)	Neurodevelopmental impairment	74.9 (12.6)	80.3 (12.2)	0.016
		≤26 weeks’ gestation	77.0 (10.6)	78.6 (14.6)	0.486
		More deprived	75.6 (14.3)	80.3 (9.8)	0.033
Self esteem	82.1 (14.0)	Neurodevelopmental impairment	80.6 (13.8)	83.3 (14.2)	0.279
		≤26 weeks’ gestation	80.5 (14.0)	83.7 (14.0)	0.199
		More deprived	82.8 (14.3)	81.2 (13.8)	0.532
Parental impact—emotional‡	70.3 (26.9)	Neurodevelopmental impairment	60.3 (29.0)	79.4 (21.3)	<0.001
		≤26 weeks’ gestation	67.2 (28.7)	73.9 (24.5)	0.111
		More deprived	69.4 (28.8)	71.5 (24.7)	0.506
Parental impact—time†	100.0 (77.8, 100.0)	Neurodevelopmental impairment	88.9 (66.7, 100.0)	100.0 (88.9, 100.0)	0.013
		≤26 weeks’ gestation	100.0 (77.8, 100.0)	94.4 (77.8, 100.0)	0.528
		More deprived	94.4 (77.8, 100.0)	100.0 (77.8, 100.0)	0.295
General health perceptions	68.2 (18.6)	Neurodevelopmental impairment	65.5 (20.6)	70.7 (16.4)	0.122
		≤26 weeks’ gestation	66.2 (18.9)	70.5 (18.3)	0.190
		More deprived	66.7 (19.5)	70.0 (17.6)	0.321
Family activities†	87.5 (66.7, 100.0)	Neurodevelopmental impairment	72.9 (54.2, 91.7)	95.8 (87.5, 100.0)	<0.001
		≤26 weeks’ gestation	87.5 (62.5, 100.0)	89.6 (70.8, 100.0)	0.602
		More deprived	87.5 (66.7, 100.0)	91.7 (70.8, 100.0)	0.137
Family cohesion	81.7 (18.6)	Neurodevelopmental impairment	79.3 (20.6)	84.0 (16.3)	0.160
		≤26 weeks’ gestation	83.7 (17.1)	79.6 (20.0)	0.218
		More deprived	79.6 (21.0)	84.2 (15.0)	0.162
Change in health	3.6 (0.9)	Neurodevelopmental impairment	3.7 (0.9)	3.6 (0.9)	0.524
		≤26 weeks’ gestation	3.6 (0.9)	3.7 (0.9)	0.405
		More deprived	3.7 (1.0)	3.6 (0.9)	0.680
Physical summary score	50.8 (11.1)	Neurodevelopmental impairment	48.0 (13.1)	53.3 (8.2)	0.009
		≤26 weeks’ gestation	49.2 (13.3)	52.6 (7.6)	0.071
		More deprived	49.5 (12.2)	52.3 (9.5)	0.151
Psychosocial summary score	49.3 (9.1)	Neurodevelopmental impairment	45.9 (9.5)	52.3 (7.6)	<0.001
		≤26 weeks’ gestation	48.5 (9.1)	50.1 (9.2)	0.333
		More deprived	48.0 (10.0)	50.7 (7.9)	0.093
**Health Utilities Index Mark 2**					
Sensation†	1.00 (1.00, 1.00)	Neurodevelopmental impairment	1.00 (1.00, 1.00)	1.00 (1.00, 1.00)	0.004
		≤26 weeks’ gestation	1.00 (1.00, 1.00)	1.00 (1.00, 1.00)	0.724
		More deprived	1.00 (1.00, 1.00)	1.00 (1.00, 1.00)	0.024
Mobility†	1.00 (1.00, 1.00)	Neurodevelopmental impairment	1.00 (1.00, 1.00)	1.00 (1.00, 1.00)	0.054
		≤26 weeks’ gestation	1.00 (1.00, 1.00)	1.00 (1.00, 1.00)	0.040
		More deprived	1.00 (1.00, 1.00)	1.00 (1.00, 1.00)	0.420
Emotion†	1.00 (1.00, 1.00)	Neurodevelopmental impairment	1.00 (0.86, 1.00)	1.00 (1.00, 1.00)	0.186
		≤26 weeks’ gestation	1.00 (0.86, 1.00)	1.00 (1.00, 1.00)	0.036
		More deprived	1.00 (1.00, 1.00)	1.00 (1.00, 1.00)	0.632
Cognition†	1.00 (0.86, 1.00)	Neurodevelopmental impairment	0.86 (0.86, 1.00)	1.00 (1.00, 1.00)	<0.001
		≤26 weeks’ gestation	1.00 (0.86, 1.00)	1.00 (0.86, 1.00)	1.000
		More deprived	1.00 (0.86, 1.00)	1.00 (0.86, 1.00)	0.310
Selfcare†	1.00 (1.00, 1.00)	Neurodevelopmental impairment	1.00 (1.00, 1.00)	1.00 (1.00, 1.00)	0.001
		≤26 weeks’ gestation	1.00 (1.00, 1.00)	1.00 (1.00, 1.00)	0.443
		More deprived	1.00 (1.00, 1.00)	1.00 (1.00, 1.00)	0.714
Pain†	1.00 (0.95, 1.00)	Neurodevelopmental impairment	1.00 (0.95, 1.00)	1.00 (1.00, 1.00)	0.214
		≤26 weeks’ gestation	1.00 (1.00, 1.00)	1.00 (0.95, 1.00)	0.723
		More deprived	1.00 (1.00, 1.00)	1.00 (0.95, 1.00)	0.723
Dead healthy†	0.94 (0.88, 1.00)	Neurodevelopmental impairment	0.92 (0.83, 0.96)	1.00 (0.92, 1.00)	<0.001
		≤26 weeks’ gestation	0.94 (0.84, 1.00)	0.96 (0.92, 1.00)	0.108
		More deprived	0.94 (0.85, 1.00)	0.96 (0.90, 1.00)	0.258

Unless stated otherwise, values are mean (SD) and analysis performed using two-tailed between-subjects t-tests. p-values are for comparison between subgroups with and without the risk factor. Neurodevelopmental impairment: “Yes” n = 60; “No” n = 67. ≤26 weeks’ gestation: “Yes” n = 67; “No” n = 60. More deprived: “Yes” n = 68; “No” n = 59. More deprived: New Zealand Deprivation Index 2013 >5.

†Values are median (interquartile range) and analyzed using Mann-Whitney U Test.

‡Square-root transformed.

Compared to their peers without NDI, participants with NDI had poorer HRQoL, with significantly lower physical and psychosocial summary scores (Table 2). NDI was associated with a higher incidence of aggressive or immature behavior and reduced physical functioning, both of which limited social opportunities, as well as increased feelings of anxiety or depression. Family dynamics were also impacted: participants with NDI interrupted family activities and restricted time for caregiver self-care more than participants without, generating greater feelings of worry or concern in caregivers.

Participants born at earlier gestational ages or experiencing higher socio-economic deprivation were less capable of performing vigorous physical tasks than their peers and were more socially limited due to emotional or behavioral problems (Table 2). Additionally, higher deprivation was associated with greater feelings of anxiety or depression in participants. However, these variables were not related to reduced family functioning.

### HUI-2

Similar to the CHQ-PF50, the HUI-2 demonstrated that NDI and, to a lesser extent, earlier gestation at birth and increased socio-economic deprivation were associated with poorer HRQoL (Table 2). Participants with NDI experienced greater cognitive difficulties than those without and were less capable of performing selfcare tasks such as eating or dressing. NDI and increased deprivation were also associated with greater sensory deficits. Earlier gestation at birth was associated with decreased physical mobility and increased feelings of irritability or anger.

### Subgroup analysis

When participants were stratified into NDI severity subgroups, both mild and severe NDI were significantly associated with reduced HRQoL (Table 3). Participants with mild NDI were more psychosocially limited than participants without NDI, and experienced poorer functioning in sensory and selfcare domains. Participants with severe NDI were further limited, scoring lower across a range of physical, mental, and social domains when compared to participants without NDI. Regarding family functioning, participants with either mild or severe NDI significantly disrupted family activities and generated caregiver stress relative to peers without NDI, with severe NDI having a greater effect than mild.

**Table 3:** Health-related quality of life in children with severe, mild, and no neurodevelopmental impairment.

Assessment	Domain	Summary score			
		Severe NDI	Mild NDI	No NDI	p
		n = 22	n = 38	n = 67	
Child Health Questionnaire Parent Form 50 questions	Physical functioning†	94.4 (59.7, 100.0)**	100.0 (88.9, 100.0)	100.0 (94.4, 100.0)	0.007
	Social limitations—emotional†	77.8 (33.3, 100.0)***	88.9 (52.8, 100.0)***	100.0 (100.0, 100.0)	<0.001
	Social limitations—physical†	100.0 (33.3, 100.0)***	100.0 (83.3, 100.0)	100.0 (100.0, 100.0)	0.001
	Bodily pain/discomfort†	100.0 (67.5, 100.0)	100.0 (80.0, 100.0)	100.0 (80.0, 100.0)	0.870
	Behaviour	61.3 (16.6)**	67.6 (20.3)	75.5 (15.7)	0.002
	Mental health	75.9 (9.6)	74.3 (14.2)	80.3 (12.2)	0.049
	Self esteem	78.0 (15.1)	82.1 (13.0)	83.3 (14.2)	0.309
	Parental impact—emotional‡	54.5 (27.7)***	63.6 (29.7)**	79.4 (21.3)	<0.001
	Parental impact—time†	88.9 (66.7, 100.0)	88.9 (75.0, 100.0)	100.0 (88.9, 100.0)	0.046
	General health perceptions	59.6 (22.4)*	68.9 (19.0)	70.7 (16.4)	0.052
	Family activities†	66.7 (39.6, 88.5)***	77.1 (61.5, 95.8)***	95.8 (87.5, 100.0)	<0.001
	Family cohesion	77.7 (20.9)	80.1 (20.7)	84.0 (16.3)	0.324
	Change in health	3.6 (1.0)	3.7 (0.8)	3.6 (0.9)	0.750
	Physical summary score	43.9 (16.8)**	50.4 (10.0)	53.3 (8.2)	0.002
	Psychosocial summary score	44.9 (8.0)**	46.4 (10.3)**	52.3 (7.6)	<0.001
Health Utilities Index Mark 2	Sensation†	1.00 (1.00, 1.00)	1.00 (0.87, 1.00)**	1.00 (1.00, 1.00)	0.006
	Mobility†	1.00 (0.92, 1.00)***	1.00 (1.00, 1.00)^*§*^	1.00 (1.00, 1.00)	<0.001
	Emotion†	1.00 (0.97, 1.00)	1.00 (0.86, 1.00)	1.00 (1.00, 1.00)	0.338
	Cognition†	0.86 (0.66, 1.00)***	0.86 (0.86, 1.00)***	1.00 (1.00, 1.00)	<0.001
	Selfcare†	1.00 (0.96, 1.00)**	1.00 (1.00, 1.00)*	1.00 (1.00, 1.00)	0.004
	Pain†	1.00 (0.95, 1.00)	1.00 (0.95, 1.00)	1.00 (1.00, 1.00)	0.429
	Dead healthy scale†	0.90 (0.82, 1.00)**	0.94 (0.83, 0.96)***	1.00 (0.92, 1.00)	<0.001

Unless stated otherwise, values are mean (SD), with analysis between groups performed using one-way ANOVA and post-hoc analyses with Tukey’s Honest Significant Difference test. P-values are for comparisons across all three groups (severe vs mild vs no NDI) without post-hoc adjustment. NDI: Neurodevelopmental impairment.

†Values are median (interquartile range) with analysis between groups performed using Kruskal-Wallis ANOVA and post-hoc analyses with Steel-Dwass test.

‡Square-root transformed.

Post-hoc analyses

*p<0.05 compared to “No NDI”.

**p<0.01 compared to “No NDI.

***p<0.001 compared to “No NDI”.

^§^p<0.01 compared to “Severe NDI”.

### Regression analyses

On univariable analyses, FSIQ and MABC-2 total score percentiles were positively related to overall HRQoL, and NZDep2013 was negatively related to overall HRQoL, but gestational age at birth was not related to HRQoL (Table 4). On multivariable analyses including FSIQ, MABC-2, and NZDep2013 in the model, FSIQ was independently predictive of physical summary score, while both FSIQ and MABC-2 total score percentiles were independently predictive of psychosocial summary score and dead-healthy scale scores. Variance inflation factors showed no evidence of collinearity between FSIQ, MABC-2, and NZDep2013 (i.e. all were <10).

**Table 4:** Relationships between measures of health-related quality of life and full-scale intelligence quotient, Movement Assessment Battery for Children total score percentile, gestational age, and socio-economic deprivation.

Regression analysis	Independent variable	Physical summary score†			Psychosocial summary score†			Dead healthy scale‡		
		Standardized β [95% CI]	r^2^	p	Standardized β [95% CI]	r^2^	p	Standardized β [95% CI]	r^2^	p
Univariable	WISC-IV full scale intelligence quotient	0.463 [0.306, 0.620]	0.214	<0.001	0.395 [0.232, 0.557]	0.156	<0.001	0.474 [0.318, 0.630]	0.225	<0.001
	MABC-2 total score percentile	0.226 [0.053, 0.398]	0.051	0.011	0.339 [0.172, 0.505]	0.115	<0.001	0.365 [0.200, 0.530]	0.133	<0.001
	Gestational age	0.109 [-0.067, 0.285]	0.012	0.223	0.090 [-0.087, 0.266]	0.008	0.316	0.081 [-0.095, 0.258]	0.007	0.365
	New Zealand Deprivation Index 2013	-0.188 [-0.362, -0.014]	0.035	0.034	-0.217 [-0.390, -0.045]	0.047	0.014	-0.193 [-0.367, -0.020]	0.037	0.030
Multivariable	WISC-IV full scale intelligence quotient	0.447 [0.253, 0.642]	0.144	<0.001	0.250 [0.053, 0.447]	0.049	0.013	0.364 [0.174, 0.554]	0.105	<0.001
	MABC-2 total score percentile	0.011 [-0.171, 0.193]	<0.001	0.903	0.214 [0.030, 0.398]	0.041	0.023	0.189 [0.011, 0.367]	0.035	0.038
	New Zealand Deprivation Index 2013	-0.028 [-0.200, 0.143]	<0.001	0.744	-0.119 [-0.293, 0.054]	0.015	0.175	-0.056 [-0.223, 0.111]	0.004	0.511

Adjusted r^2^ for physical summary score = 0.196. Adjusted r^2^ for psychosocial summary score = 0.178. Adjusted r^2^ for dead healthy scale = 0.234. WISC-IV: Wechsler Intelligence Scale for Children 4^th^ Edition. MABC-2: Movement Assessment Battery for Children 2^nd^ Edition. CI: confidence intervals. New Zealand Deprivation Index 2013 scored from 0–10, with higher scores indicating greater socio-economic deprivation.

†From Child Health Questionnaire Parent Form 50 Questions.

‡From Health Utilities Index Mark 2.

## Discussion

Our research objective was to assess caregiver reported HRQoL in a cohort of New Zealand children born <30 weeks’ gestation or <1500 g birthweight, and describe the relationship with NDI, gestation at birth, and socio-economic deprivation. We found that at 7 years’ corrected age, caregivers reported good overall HRQoL for children born very and extremely preterm on both the CHQ-PF50 and HUI-2 questionnaires. However, NDI was associated with reduced HRQoL across multiple domains, significantly impacting both individual and family functioning. Lower gestation at birth and increased socio-economic deprivation were also associated with reduced individual functioning, although these effects appeared to be largely due to NDI.

Our cohort had good overall HRQoL at 7 years, consistent with a 2016 study that reported similar findings in healthy preterm Finnish children born very low birthweight [19]. However, another study from France reported that extremely preterm children have poorer overall HRQoL than full-term peers [20]. Our study also found that caring for children with NDI significantly reduced family HRQoL, most noticeably in caregiver emotional states. Others have also reported that caregivers of hospitalized extremely preterm infants experience more negative emotions than population norms [21]. This may have long-term implications for their psychological wellbeing and contribute towards the increased emotional distress we observed.

The incidence of NDI in our cohort was higher than reported in other studies of children born very preterm [22]. This may be partly because the incidence and severity of NDI increases as gestational age decreases, and 71% of our cohort was born extremely preterm. Additionally, we used a broader definition of NDI to capture participants with both mild and severe impairment, to further explore the relationship between NDI and HRQoL. Commonly, studies on extremely preterm children focus on severe NDI, which is likely to more strongly influence a child’s future functioning and HRQoL than mild NDI; a pattern which our findings support. However, our study demonstrates that mild NDI is not only more common than severe NDI, making up 63% of our NDI subgroup, but is also an important contributor to reduced psychosocial HRQoL in later childhood.

Given the propensity of NDI to interfere with multiple domains of childhood functioning, the mechanism by which it influences HRQoL is complex. The third trimester of pregnancy is a critical period for brain development, and preterm birth is associated with disrupted neural connections and altered brain structure [7]. These in turn are related to specific neurological abnormalities which limit physical and psychosocial functioning and can impact HRQoL. Furthermore, these functional limitations may prevent or discourage children from engaging in certain social activities, changing their social environment which can further influence psychosocial outcomes [6].

It remains controversial whether poorer psychosocial outcomes after preterm birth can be attributed to an increased prevalence of internalizing or externalizing behaviors [23]. However, a 2014 study found that neonatal procedural pain contributed towards increased internalizing behavior at 7 years [24], which can restrict social opportunities. Children born preterm, especially those with NDI, may also be shyer due to pre-existing physical or intellectual issues, which can become more visible in the school environment [25]. These are consistent with the so-called preterm behavioral phenotype, which identifies preterm children collectively as being at greater risk for anxiety, inattentive attention-deficit/hyperactivity disorder, and autism spectrum disorder associated with neurological etiologies, although it should be noted that most children born extremely preterm have minimal behavioral difficulties [26]. Our findings are consistent with these reports. While our cohort got along well with others overall, children born at earlier gestations were more socially limited due to emotional and behavioral issues than children born later. However, it should be noted that the CHQ-PF50 focuses specifically on measuring externalizing rather than internalizing behavioral HRQoL.

Socio-economic deprivation has also been shown to influence HRQoL, with previous studies reporting an association between increased deprivation and poorer perceptions of health, emotional and behavioral issues, and NDI [5, 8, 27]. Stress associated with low socio-economic status and reduced access to social resources have been proposed as potential mediators for this relationship.

Children born very preterm are more likely than full-term peers to experience motor impairment, including both cerebral palsy and non-cerebral palsy manifestations [28, 29]. The incidence of non-cerebral palsy motor problems has been reported to be as high as 28% in school-aged children born very preterm [28, 30], and screening has been recommended at or before preschool age to identify motor difficulties so that interventions can be administered in a timely fashion [29, 31]. Our study findings support this concern: motor impairment was common in our cohort, with 44 (35%) children having MABC-2 scores ≤15^th^ percentile and 34 (27%) having scores ≤5^th^ percentile, although only 10 (8%) had a diagnosis of cerebral palsy.

On multivariable analysis, we found that intelligence quotient (IQ) was an independent predictor of overall physical and psychosocial HRQoL. This may be related to caregiver perceptions; if the caregiver is aware that their child has reduced IQ, they may assume that their child is unable to participate in certain physical or social activities, and so interpret that as reduced physical or psychosocial HRQoL. Furthermore, as a group, preterm children with reduced IQ are more likely to have motor impairment [32]. This was also evident in our cohort where 26 (63%) of the 41 children with reduced IQ (FSIQ <85) also had reduced motor function (MABC-2 ≤15^th^ percentile). We also found that MABC-2 was an independent predictor of psychosocial but not physical HRQoL. While perhaps initially surprising, this finding is consistent with the social model of disability, which argues that socio-political rather than exclusively biomedical factors are a major driver of poor HRQoL in people with disabilities [33]. Indeed, it has been suggested that “some of the most restricting and debilitating features in the lives of disabled people… [are] socially and politically constructed” rather than a consequence of the disability itself [34]. In addition, children with physical disabilities may not be able to participate in some activities such as sports, which may restrict their social opportunities [35].

Our study was limited by its small sample size. Further, since the eligible cohort were specifically selected on the basis of neonatal events [10] and we have no data on the characteristics of those who were not selected, or those who were selected but were not able to be assessed, we are unable to determine the generalisability of our findings. Additionally, we measured participant HRQoL indirectly through caregivers, who have been shown to both under- and over-report HRQoL, although this effect varies between different measures and domains [36]. However, both the CHQ-PF50 and HUI-2 are widely used measures of HRQoL, and their use in pediatric outcome studies have been supported by previous research [15]. Nonetheless, future research into the HRQoL of children born preterm should utilise both caregiver-reported and self-reported measures in order to more fully understand participant wellbeing [37].

In summary, we found that caregivers report good HRQoL in a cohort of New Zealand children born <30 weeks gestation or <1500g birthweight. Although NDI, lower gestational age, and greater socio-economic deprivation are all associated with lower HRQoL, only IQ and motor function are independent predictors of overall HRQoL. Strategies to improve HRQoL in children born very preterm need to continue to focus on prevention of NDI.
